# Supplementary material for: Teaching Communication and Functional Life Skills in Children Diagnosed with Autism Spectrum Disorder
Source: Behav Sci (Basel). 2025 Feb 12;15(2):198. doi: 10.3390/bs15020198 (PMC11852024; doi:10.3390/bs15020198)
Supplement: Supplementary file 1 [file behavsci-15-00198-s001.zip › behavsci-3407536-supplementary.pdf]

Behavioral sequences

Cleaning up the hands

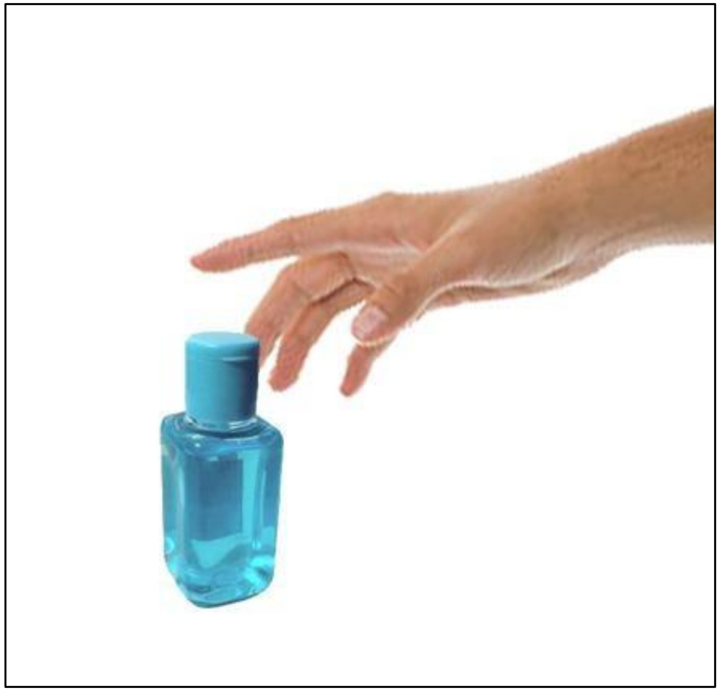

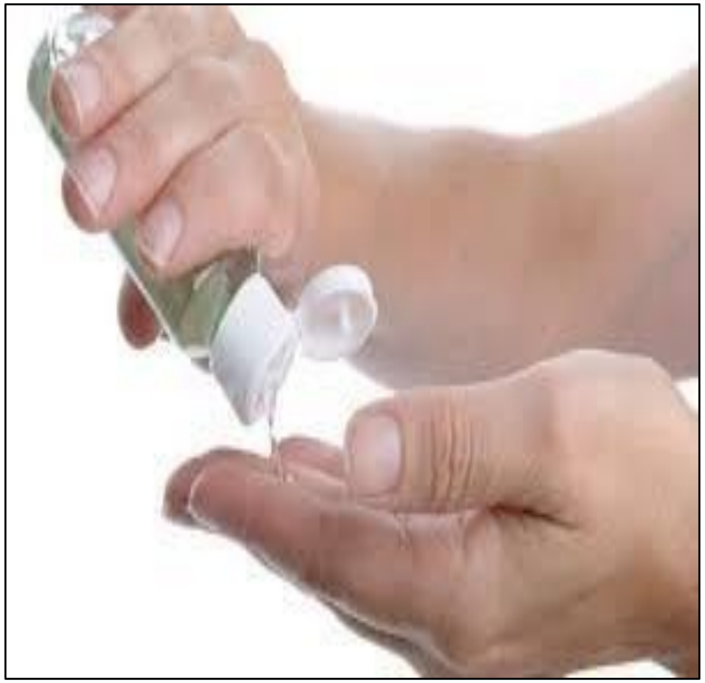

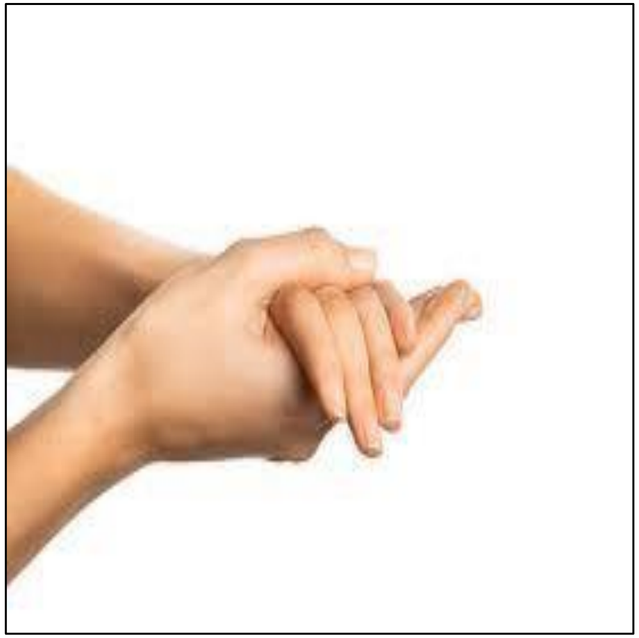

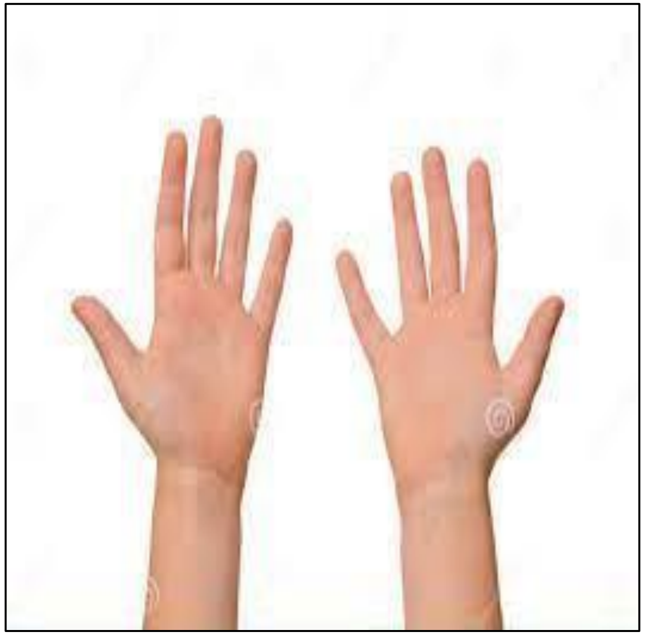

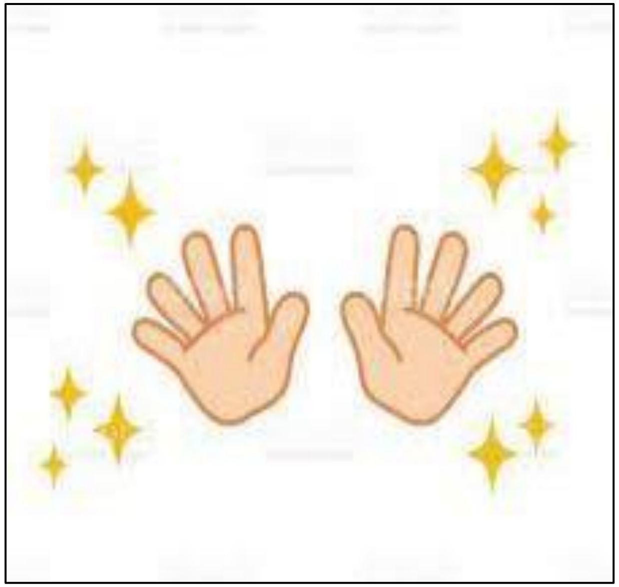

Putting on the mask and keeping it on the face

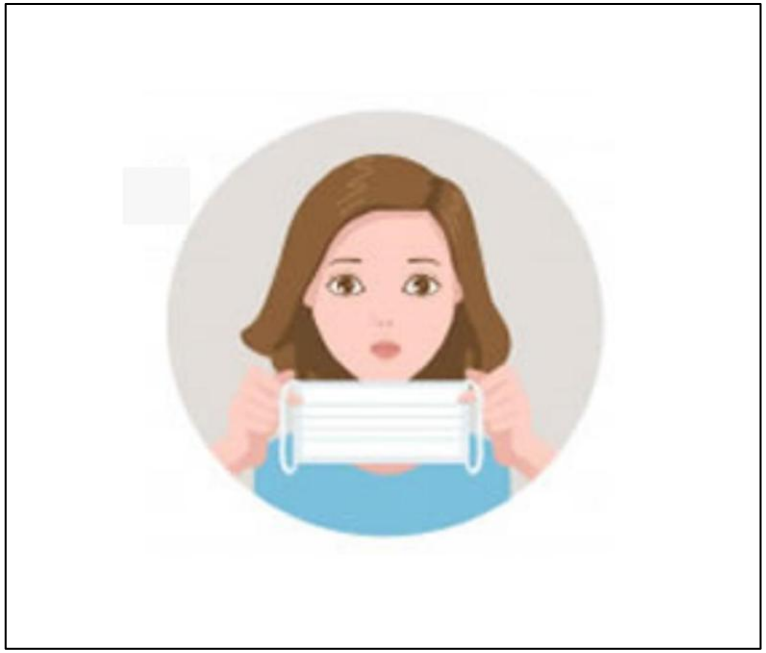

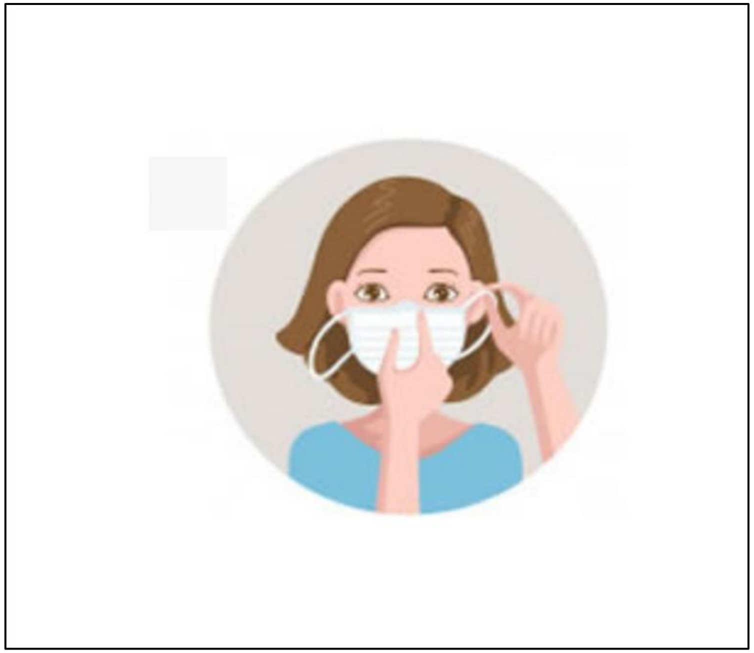

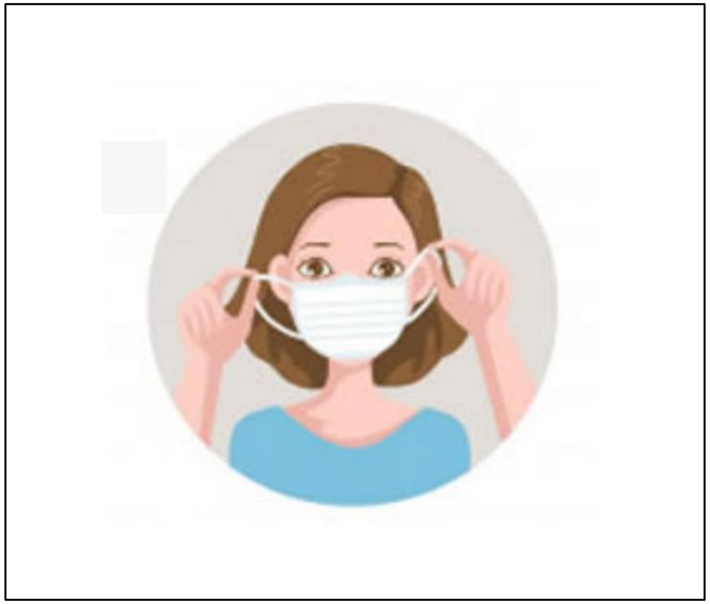

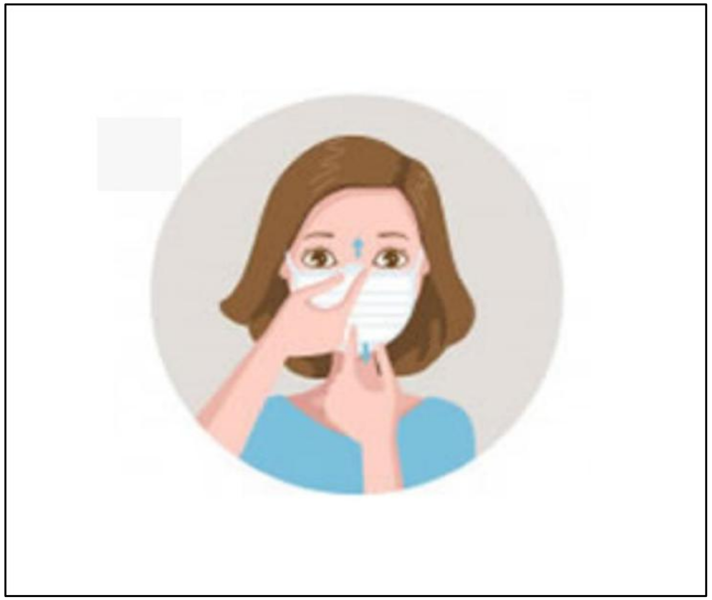

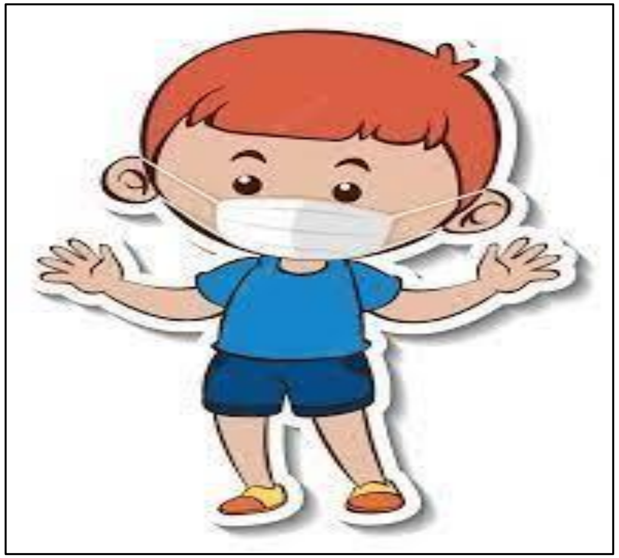

Functional play (Mr. Potato Head doll)

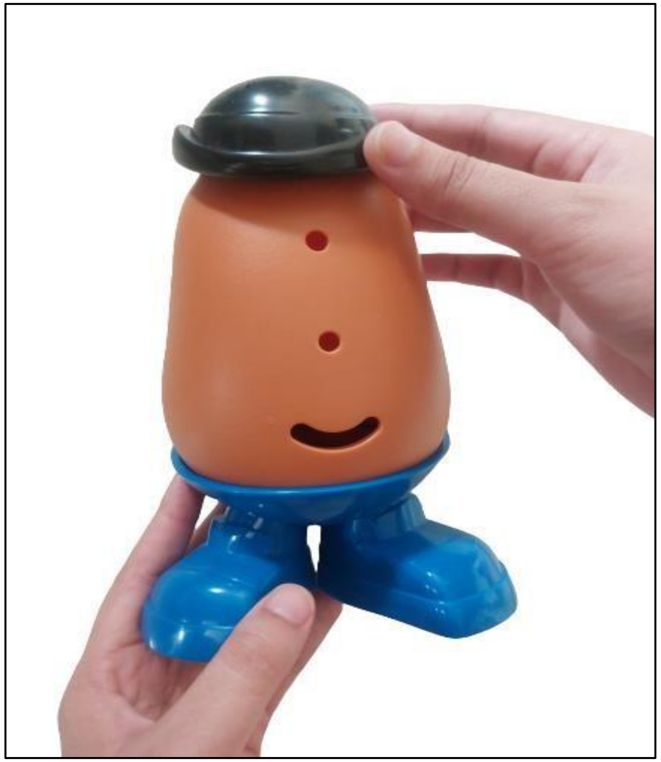

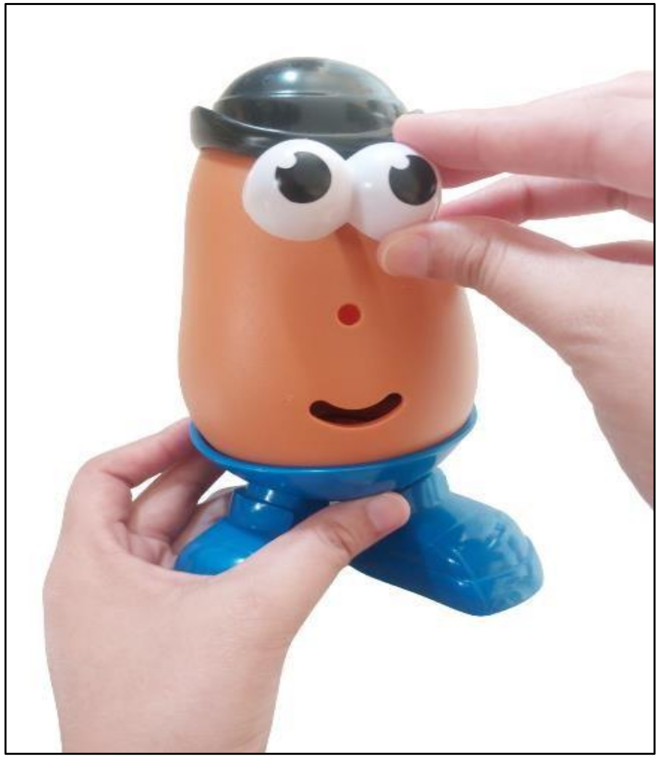

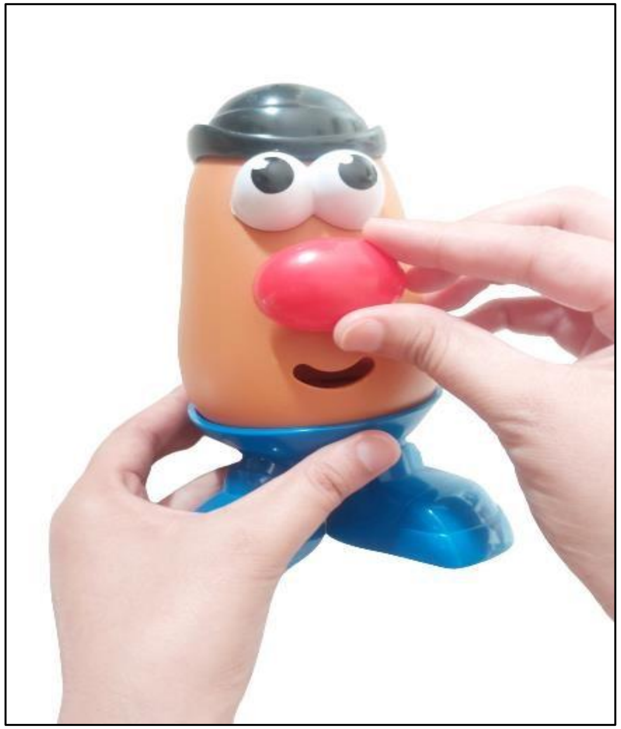

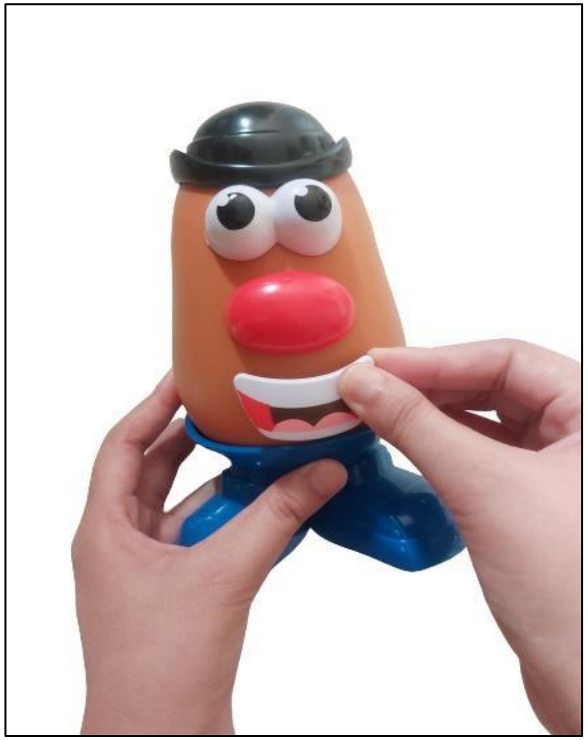

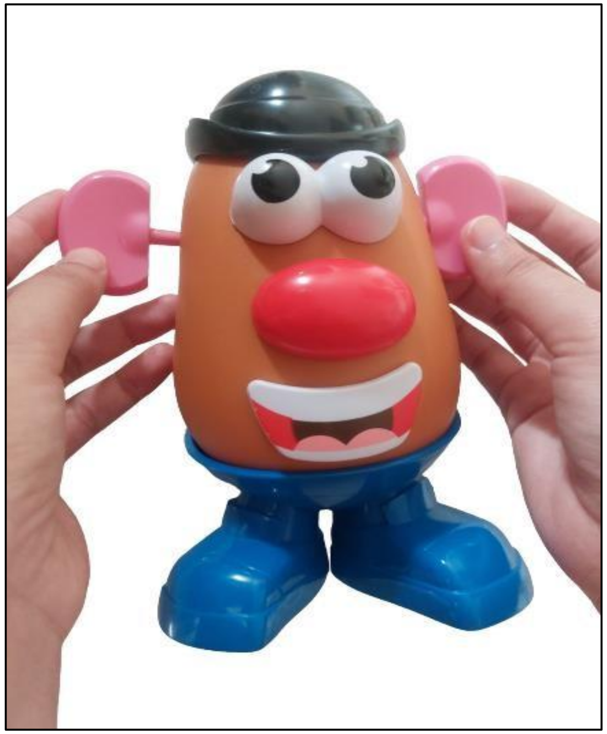

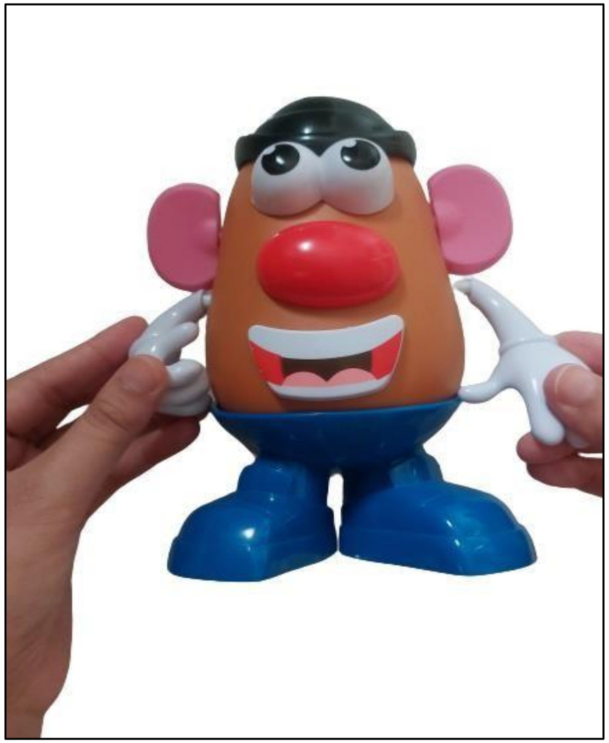

Brushing teeth

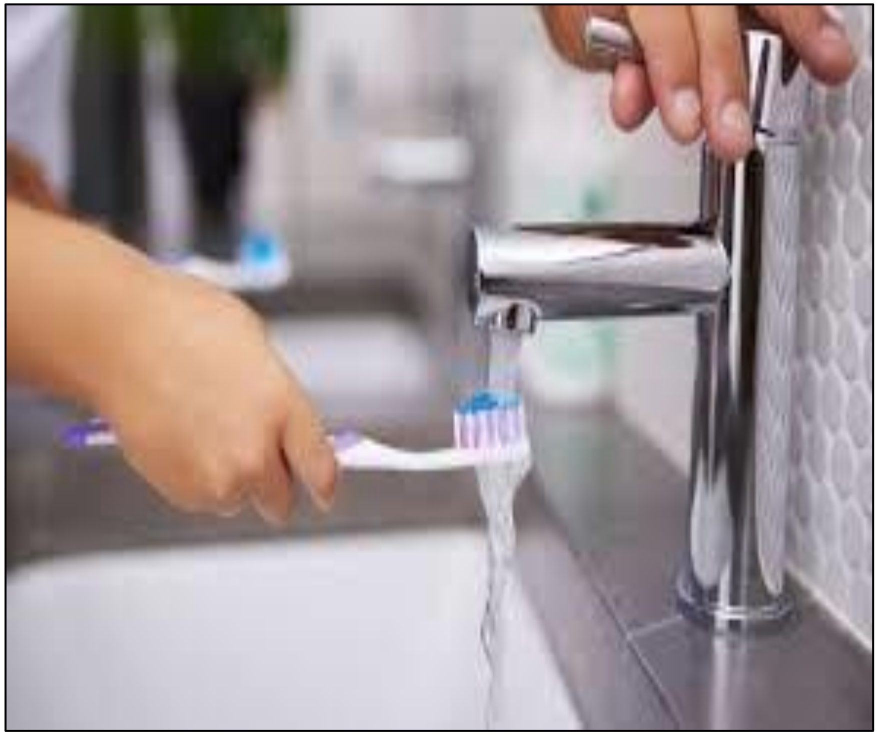

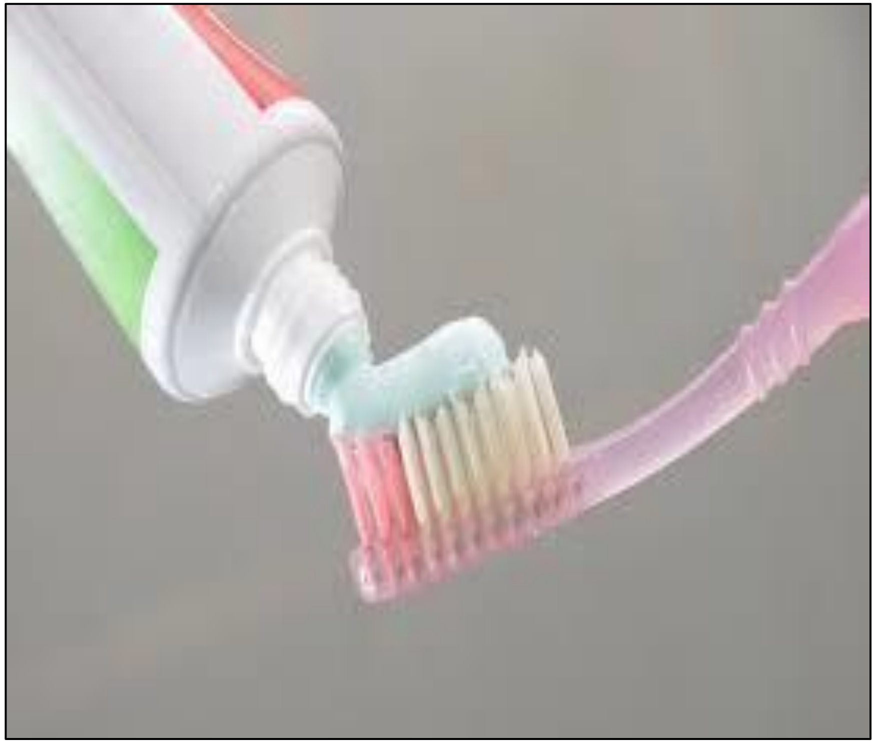

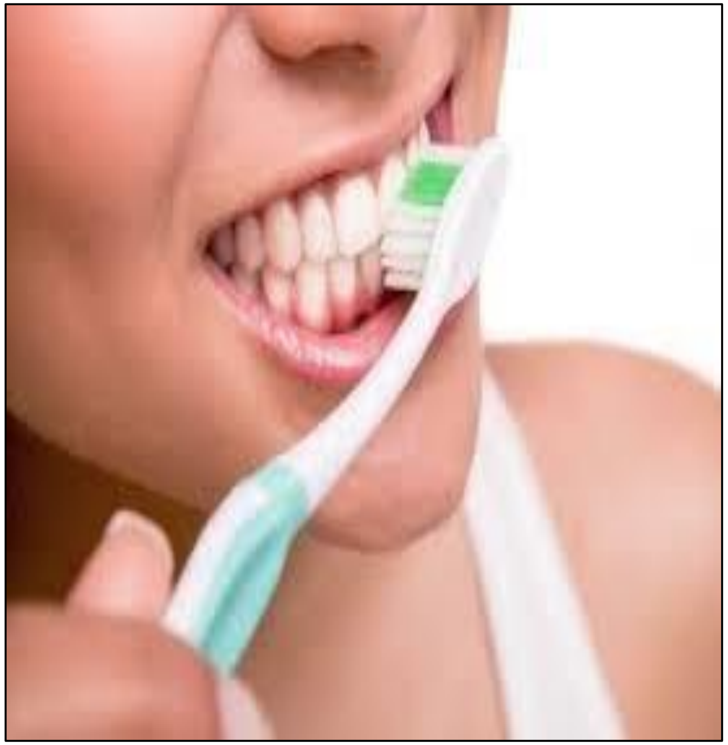

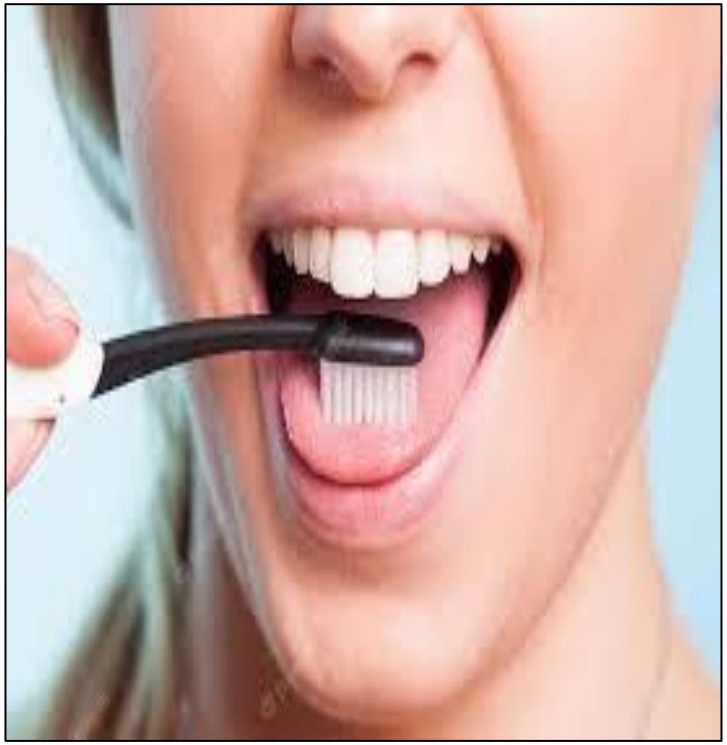

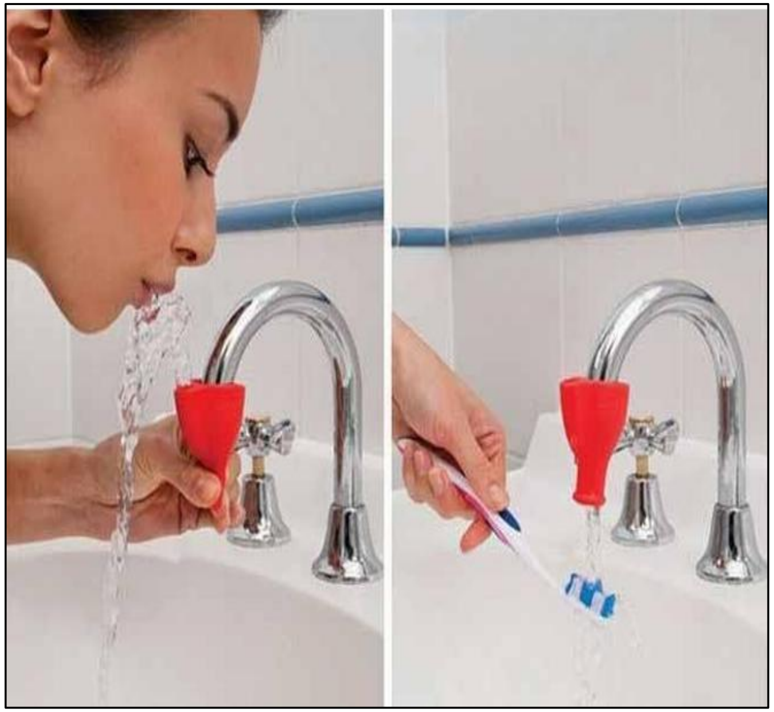

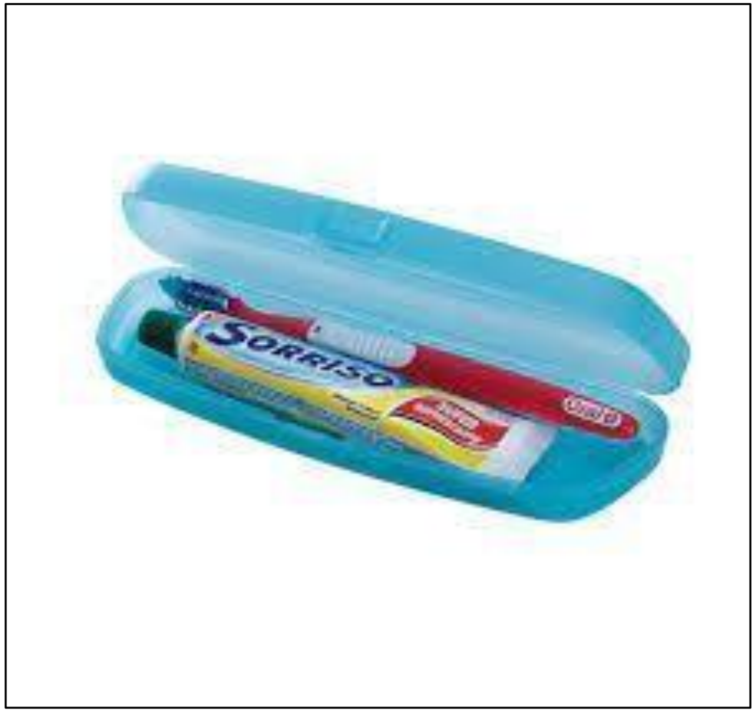
SORRISO
